# Excess direct hospital cost of treating adult patients with ventilator associated respiratory infection (VARI) in Vietnam

**DOI:** 10.1371/journal.pone.0206760

**Published:** 2018-10-31

**Authors:** Vu Quoc Dat, Vu Thi Lan Huong, Hugo C. Turner, Louise Thwaites, H. Rogier van Doorn, Behzad Nadjm

**Affiliations:** 1 Department of Infectious Diseases, Hanoi Medical University, Hanoi, Vietnam; 2 Wellcome Trust Asia Programme, Oxford University Clinical Research Unit, Hanoi, Vietnam; 3 Nuffield Department of Clinical Medicine, Centre for Tropical Medicine and Global Health, University of Oxford, Oxford, United Kingdom; University of Notre Dame Australia, AUSTRALIA

## Abstract

**Introduction:**

Ventilator associated respiratory infections (VARIs) are the most common hospital acquired infections in critical care worldwide. This work aims to estimate the total annual direct hospital cost of treating VARI throughout Vietnam.

**Methods:**

A costing model was constructed to evaluate the excess cost of diagnostics and treatment of VARI in Vietnam. Model inputs included costs for extra lengths of stay, diagnostics, VARI incidence, utilisation of ventilators and antibiotic therapy.

**Results:**

With the current VARI incidence rate of 21.7 episodes per 1000 ventilation-days, we estimated 34,428 VARI episodes in the 577 critical care units in Vietnam per year. The extra cost per VARI episode was $1,174.90 and the total annual excess cost was US$40.4 million. A 1% absolute reduction in VARI incidence density would save US$1.86 million annually. For each episode of VARI, the share of excess cost components was 45.1% for critical care unit stay and ventilation, 3.7% for diagnostics and 51.1% for extra antimicrobial treatment.

**Conclusions:**

At the current annual government health expenditure of US$117 per capita, VARI represents a substantial cost to the health service in Vietnam. Enhanced infection prevention and control and antimicrobial stewardship programmes should be implemented to reduce this.

## Introduction

Hospital acquired infections (HAIs) are a major concern in healthcare institutions world-wide. HAI prevalence ranges from 4% in the United States [1] to 7.1% in Europe [2]. In a literature review of 6 countries from 41 studies in Southeast Asia between 2000 and 2012, HAI occurred in 20 patients per 1000 intensive care unit-days with the highest incidence density attributed to ventilator-associated pneumonia (14.7 per 1000 ventilator-days) [3]. In critical care units (CCUs), approximately 30% of patients acquire HAI during their stay: [4] the most common being ventilator-associated pneumonia (VAP) and ventilator-associated tracheobronchitis (VAT). VAP and VAT can be grouped together as Ventilator Associated Respiratory Infections (VARI), a term that then encompasses the spectrum of respiratory infections that clinicians in Vietnam and many parts of the world treat with antibiotics. VARI is associated with excess length of stay, prolonged duration of ventilation, increased cost and, (in some studies) increased mortality. Incidence densities of 3.2–56.9 per 1000 ventilator-days have been reported in resource constrained setting [5].

The direct financial costs of such infections have been reported to be high [6, 7], and it is important to improve our understand of these costs in order to mobilise resources for prevention and control measures. In a systematic review of publications between 2001 and 2004, mostly in high-income settings, the mean excess cost of VAP was US$ 9,969 ± 2,920 [6]. A more recent estimate from the USA found that the average excess cost per VAP case for an adult inpatient was $40,144 (36,286–44,220) [7]. In lower and middle income countries, the case-based cost is considered significantly lower but not well described [3, 8]. However the incidence is higher [5] and a greater proportion of the costs may be met out of pocket, leading to substantial impacts on the patients and their families [9].

Vietnam is a lower-middle income country with a population of 93 million and a gross national income (GNI) per capita of US$ 2,060 in 2016 [10]. As of July 2016 Vietnam had 13,571 healthcare facilities, including 1,132 general and specialized hospitals with 305,700 in-patient beds [11, 12]. This figure includes 75 central hospitals (including 38 national hospitals) that receive referrals from outside of the local province, 502 provincial or equivalent hospitals that receive referrals from within the local province, 548 districts hospitals and 7 unclassified hospitals that receive patients only directly from the community [12].

Although in Vietnam, HAI is an increasing concern, little is known about its economic impact. High levels of antibiotic resistance [13] and infection control programmes which are often inadequate means this is increasingly important, and VARI has been shown to be a particular problem [14, 15]. This study aims to estimate the total annual excess cost associated with VARI in critical care units in Vietnam. Such an estimate will help policy makers in planning and implementing necessary changes in infection control, antibiotic stewardship and other relevant policies.

## Materials and methods

### Study approach

We estimated the excess cost of VARI in Vietnam using a incidence-based cost-of-illness approach [16] from a healthcare sector perspective which included all costs covered by the governmental health insurance and co-paid by patients. We did not distinguish between VAP and VAT as the pathogenesis and bacterial aetiology of the two entities are essentially the same and both are treated for a similar duration in Vietnam. A Vietnamese study analysing the costs of VAP and other VARI found little difference when similar patient groups were looked at [15]. All direct medical costs were estimated as the product of number of services and their unit cost. Micro-costing methodology was used to cost the management of VARI which was contributed to by cost components of diagnostics, hospital stays and antimicrobials. By using this method, the direct costs were estimated as the product of number of services used and their unit cost.

### Data resources

We used a 2015 survey of 25 provincial hospitals and 103 district hospitals in Vietnam to derive the number of hospital beds with access to mechanical ventilation in Vietnam and the proportion of these ventilators in frequent use [17]. In this survey critical care beds accounted for a median of 4% (IQR 3–5%) of the provincial hospital beds and for 9% (IQR 5–12%) of the district hospital beds. This corresponded to median critical care unit sizes of 26 beds (IQR 17–33) and 12 beds (IQR 8–19) in provincial and district levels respectively. All provincial CCUs had ventilators with a median number of mechanical ventilators of 11 (IQR 6–16 or 0.4 per actual critical care bed (IQR 0.26–0.55)). However, only 73.6% of district hospitals had ventilators with a median of 0.08 (IQR 0.00–0.17) ventilators per actual critical care bed. Whilst most provincial hospitals have adequate capacity to deliver critical care services, district hospitals have limited capability in this respect, typically only ventilating patients prior to transfer to a higher level of care [17]. We therefore include only central and provincial hospitals in modelling the burden of VARI.

**Fig 1:**
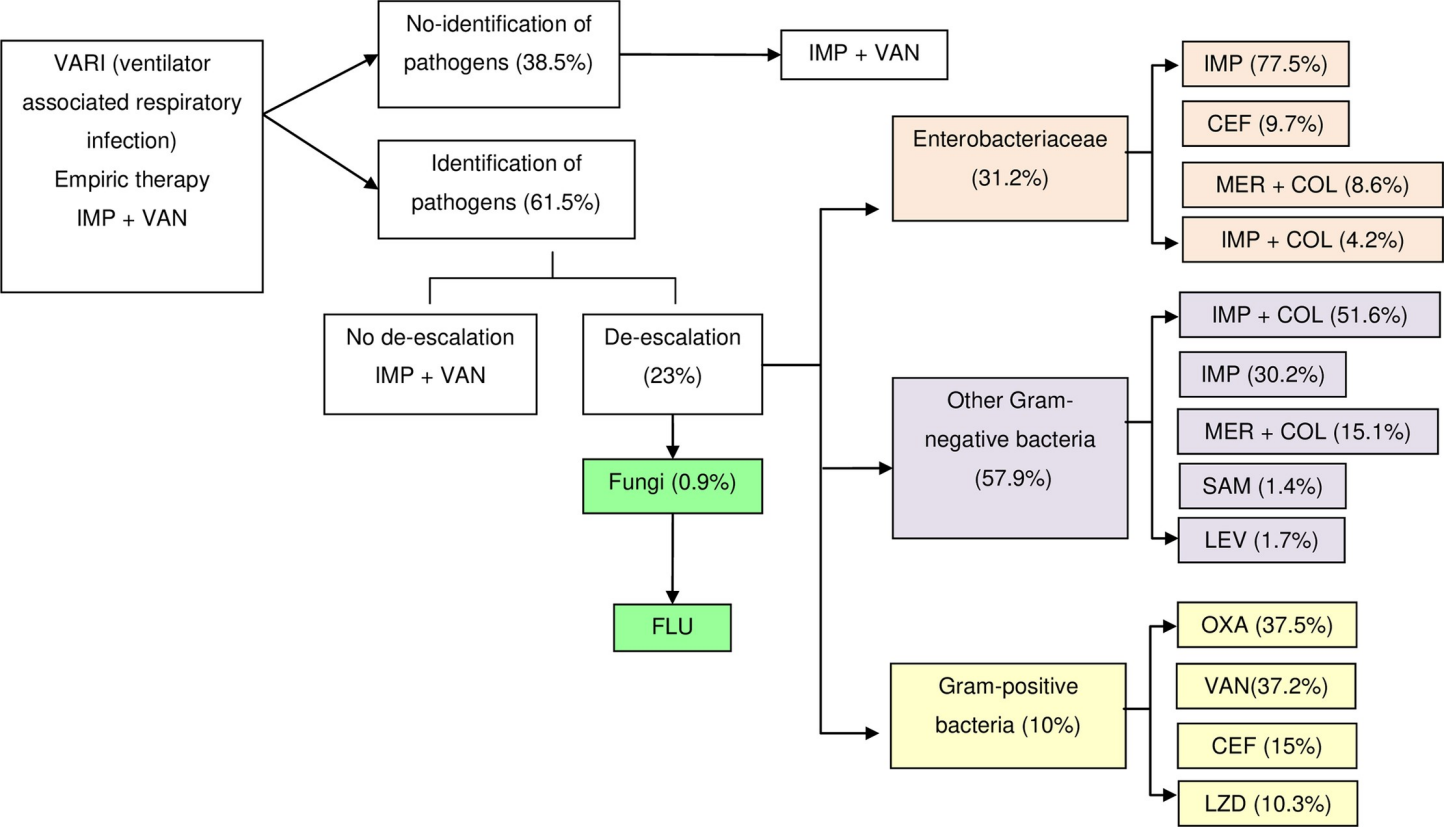
Decision tree for antibiotic treatment for ventilator associated respiratory infection (VARI). CEF, ceftriaxone; COL, colistin; FLU, fluconazole; IMP, imipenem/cilastatin; LEV, levofloxacin; LZD, linezolide; MER, meropenem; OXA, oxaciclin; SAM, ampicillin sulbactam; VAN, vancomycin.

The prevalence and associated increased length of CCU stay of VARI were extracted from literature review (Table 1). Etiology and susceptibility of VARI causing pathogens was extracted from a previous point-prevalence surveys in 15 hospitals in Vietnam (Table 1, Fig 1) [13]. Data on the unit costs of healthcare services in Vietnamese CCUs were taken from documents issued by the ministry of health regarding costs for medical services among hospitals at the same level across the country [18, 19]. Costs for these services are inclusive of certain direct expenses (i.e. consumable materials, substitute items), expenses for electricity, water, fuel, waste, treatment, maintenance and replacement of equipment and employee allowances and salaries [18]. Assumptions for the costing model are shown in Table 1.

**Table 1:** Assumptions of a costing model for ventilator associated respiratory infection (VARI) in Vietnam.

Assumption	Value (range)	Source(s)
Number of critical care units (hospitals)	577	[12]
Number of ventilators per critical care units	11 (6–16)	[17]
Proportion of patients with intubation ≥ 48 hours	88.9% (87.6%-90.2%)	[20]
Incidence density of VARI per 1000 ventilation days	21.7 (17.7–26.5)	[15]
Percentage of ventilators in frequent use	77% (± 10%)	[21]
The de-escalation rate	23% (21.3%-68%)	[22]
Extra CCU length of stay for each episode of VARI (days)	12 (± 2)	[15]
Extra duration of antibiotics (days)	12 (7–14)	[15, 23]
Excess duration of ventilation (days)	12 (± 2)	[15]
Range of etiologies of VARI	See Fig 1	[13]

Data on the cost of antimicrobials in Vietnam was taken from the 2017 medication bid-winning results of national and provincial hospitals in Vietnam as published on the Drug Administration of Vietnam website [24]. The bid-winning price reflects the diversity in pricing of antimicrobials across 93 national and provincial hospitals in 27 provinces in Vietnam. The average price of antimicrobials was applied for the model.

We developed a decision tree for antimicrobial treatment of VARI based on local data on the microbiological etiology of VARI and internationally recognised recommendations for antimicrobial treatment [23, 25] which is aligned with the national guidelines issued by the ministry of health for use in Vietnamese hospitals (Fig 1). The local data for aetiology and associated antibiotic susceptibility was derived from a previous point-prevalence survey in Vietnam [13] (S1 Table) and used in conjunction with the guidelines of the Infectious Diseases Society of America and the American Thoracic Society (or where the treatment recommendation was not available for specific pathogens, the Sanford guide to antimicrobial therapy) to determine the antimicrobial treatment administered [23, 26] (Fig 1).

The cost of VARI included the costs of initial empirical and targeted therapy regimens. The initial broad-spectrum empirical therapy was modelled to cover *P*. *aeruginosa* and ESBL-producing organisms, *Acinetobacter* spp. and methicillin-resistant *Staphylococcus aureus* (MRSA) for the setting with high rates of multidrug resistant pathogens [23, 25]. The empirical therapy was assumed to last for 3 days and followed by the targeted therapy (Fig 1).

Analysis was limited to adult patients because of limited data in the paediatric critical care population concerning the aetiology of VARI, provision and utilisation of critical care beds and difficulties accounting for the spectrum of ages to adjust antimicrobial doses.

### Estimation of the cost of VARI management and number of VARI episodes

Estimates of the cost of VARI episodes were based on the excess direct medical costs of diagnostics, hospital stay, ventilation and antimicrobial treatment. All costs were at 2017 prices and presented in US dollars (US$) based on the official exchange rate of 2016 (1 US$ = 21,935 Vietnam Dong) [27]. The diagnostics for VARI included investigations for VARI (complete blood count, arterial blood gas, bedside chest radiography, endotracheal aspirate smear and culture, blood culture and antibiotic susceptibility when the pathogen was identified) [23, 25]. These investigations are performed routinely in referral hospitals in Vietnam and recommended by the ministry of health for critical care units. All investigations were assumed to be performed at the time of VARI suspicion. The excess cost of hospital stay and ventilation was calculated by the quantity of extra days multiplied by their daily prices (Tables 1 and 2).

**Table 2:** Costs associated with management of ventilator associated respiratory infection (VARI) in Vietnam.

Item	Average unit cost (min-max) in USD	Notes
**Cost for hospital beds**	US$ 21.87 (30.98–12.77)	Daily cost
**Cost for mechanical ventilation**	US$ 22.35 (24.39–20.31)	Daily cost
**Cost for diagnostics of VARI**		
Complete blood cells	US$ 3.09 (1.46–4.71)	1 time
Air blood gas	US$ 9.42 (9.15–9.70)	1 time
Bedside chest Xray	US$ 2.91 (2.65–3.16)	1 time
Blood cultures	US$ 11.14 (9.15–13.13)	1 time
Endotracheal aspirate culture	US$ 11.14 (9.15–13.13)	1 time
Endotracheal aspirate smear	US$ 2.80 (2.61–3.00)	1 time
Qualitative antibiotic susceptibility	US$ 7.62 (7.09–8.14)	If the pathogen is identified
C-reactive protein (CRP)	US$ 2.36 (2.29–2.42)	1 time
**Cost for antimicrobial treatment**		
Linezolid 300 mg	US$ 25.39 (8.62–42.16)	600 mg IV q12h
Meropenem 1 g	US$ 19.29 (3.17–35.41)	1 g IV q8h
Colistimethate Sodium1 MIU	US$ 14.05 (10.48–17.62)	5 mg/kg IV × 1 (loading dose) followed by 2.5 mg × (1.5 × CrCl + 30) IV q12h
Fluconazole 200 mg	US$ 11.65 (6.56–16.74)	400 mg q12h
Imipenem + cilastatin 500/500 mg	US$ 9.46 (2.61–16.31)	500 mg IV q6h
Levofloxacin 500 mg	US$ 7.75 (1.19–14.32)	750 mg IV q24h
Vancomycin 1 g	US$ 4.16 (0.33–7.99)	15 mg/kg IV q8–12h
Ceftriaxone 1g	US$ 3.92 (2.47–5.37)	1 gm IV q24h
Ampicillin sulbactam 1g/0.5 g	US$ 1.88 (0.44–3.33)	3 g IV q6hr
Ceftazidim 1g	US$ 1.69 (0.48–2.91)	2 g IV q8h
Oxacillin 1g	US$ 1.25 (0.65–1.85)	2 gm IV q4h

The antimicrobials cost per VARI episode was composed of the cost of three days of empirical therapy with imipenem/cilastatin (IMP) and vancomycin (VAN) and the cost of targeted therapy. For VARI without pathogen identification, we assumed that the empirical treatment continued for the whole course of VARI treatment in n days:

$$C_{emperical\;antimicrobials}=n\times (C_{\mathrm{IMP}}+C_{\mathrm{VAN}})$$

For cases with pathogen identification, in the absence of local data, we assumed that the de-escalation was 23% as reported in a prospective Spanish observational study [22] and the empirical therapy remained unchanged in 77% of VAP patients. We calculated the proportion of antimicrobial regimens corresponding to the frequency of pathogenic isolates in patients with antimicrobial de-escalation therapy. The per-episode antimicrobial cost of microbiologically confirmed VARI cases was calculated as follows:

$$C_{\mathrm{targeted}\;\mathrm{antimicrobials}}=3\times (C_{\mathrm{IMP}}+C_{\mathrm{VAN}})+\sum {(n‑3)C}_{k}P_{k}$$

In which, n is the antimicrobial therapy duration (days), k is the antimicrobials regimen, C and P is the daily cost and proportion of usage of the specific regimens.

The country’s annual number of VARI episodes was estimated from the incidence density of VARI (in cases per 1000 ventilation days) multiplied by the total number of ventilators in frequent use in all critical care units and the proportion of patients intubated for more than 48 hours (see Table 1).

### Sensitivity analysis

A one-way sensitivity analysis of the total cost of VARI treatment was performed by varying the country capacity for mechanical ventilation, incidence density of VARI, cost of diagnostics, critical care stay and antimicrobials, and duration of antimicrobial therapy. We performed one-way sensitivity analyses in which each factor was individually varied within the assumed plausible ranges as described above whilst all other factors remained at the central values.

Among variables included in the costing model, the number of critical care units was considered as fixed value whilst the proportion of patients with intubation ≥ 48 hours and percentage of ventilators in frequent use were varied by ±10% from the average values. We varied the number of ventilators per critical care unit by the interquartile range as reported in the previous study in Vietnam [17]. Because the antimicrobial therapy de-escalation rate was not reported in Vietnam, we varied this rate by the values from the prospective study of 24 intensive care units in Spain [22]. Varying the incidence density of VARI was performed with the additional analysis of data from a previous prospective observational study in 4 CCUs in Vietnam [15]. The extra duration of antimicrobial therapy was varied between 7–14 days to cover 2 common antimicrobials regimens of 7–8 days and 10–14 days [25]. The excess CCU length of stay and duration of ventilation was varied by ±2 days from the difference of length of stay (12 days) between patients with VAP and no VAP in a meta-analysis (of studies largely in high income settings) and a prospective study in Vietnam [15, 28]. The range of cost values is shown in the Table 2.

## Results

**Fig 2:**
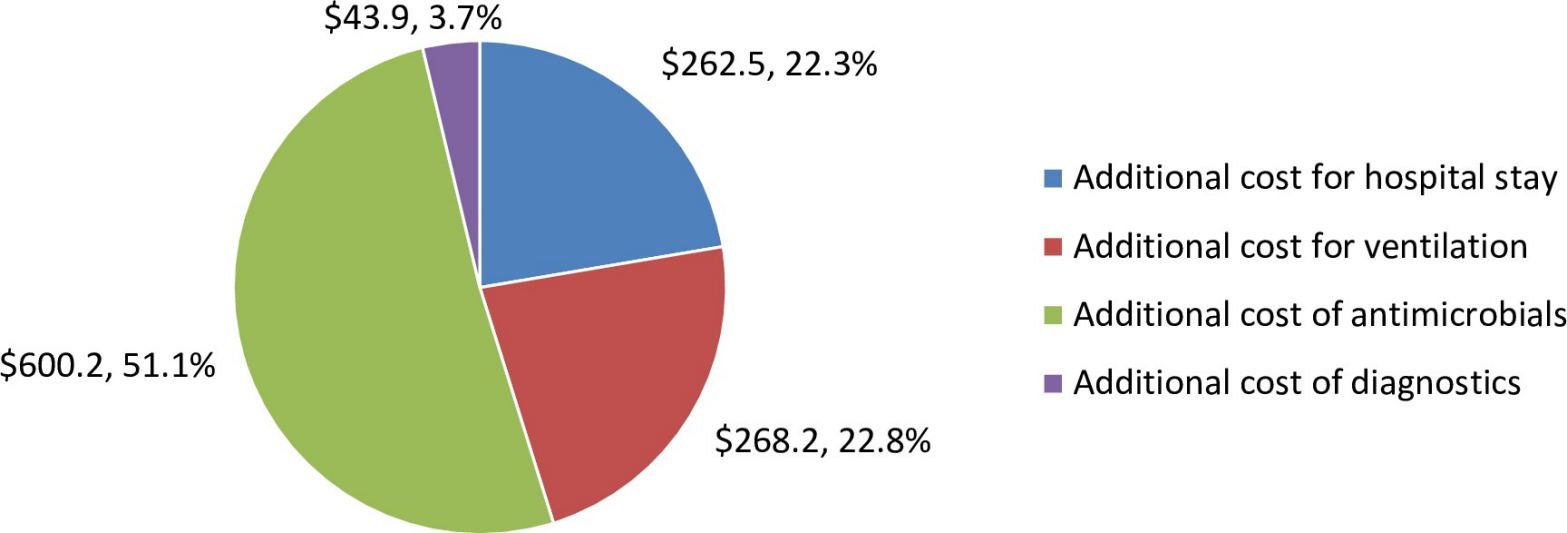
The share of cost components per VARI episode.

The average excess cost of VARI management in national and provincial hospitals in Vietnam was US$ 1,174.90 per episode (range from US$ 682.10–1,667.60) with the cost of antibiotic treatment comprising US$ 600.20 (US$ 247.60–952.90) or 51.1% of the total cost. The share of average cost components per VARI episode were shown in Fig 2.

**Fig 3:**
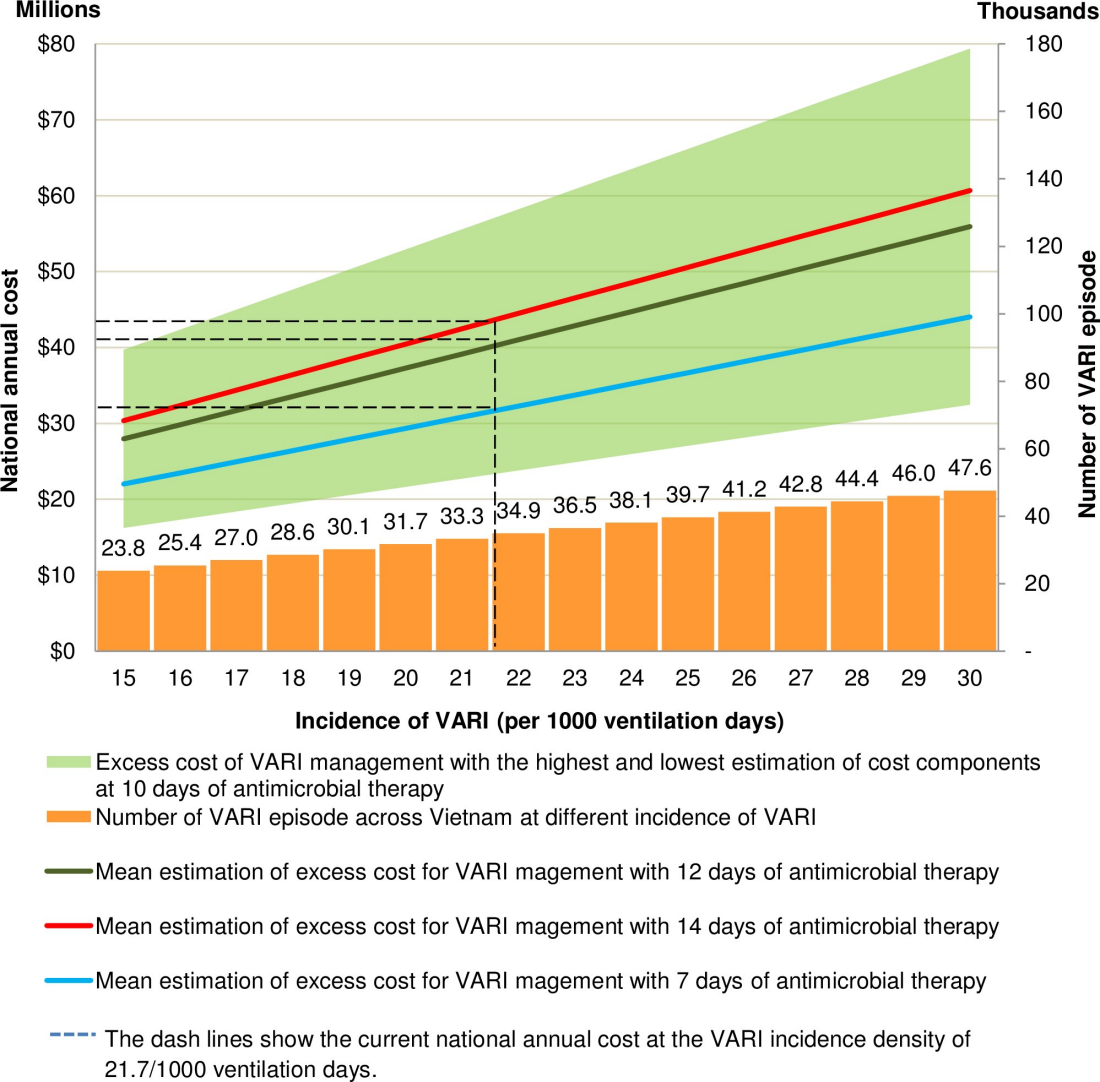
Annual excess cost for VARI in Vietnam.

We estimated the number of cases of VARI in Vietnam was 34,428 episodes annually, at a VARI incidence of 21.7/1,000 ventilation days (Fig 3). The estimated excess cost of VARI nationwide was US$ 40,447,469 (range US$ 23,482,151–57,412,787). An absolute reduction in VARI incidence density of 1% led to a decrease of 1,578 episodes per year and a saving of US$ 1.86 million nationally.

The cost of 12 days antimicrobial therapy for VARI contributed US$ 20,664,650 (US$ 8,524,541–32,804,759) to the national cost and a decrease in cost of US$ 1.72 million per one day reduction in the duration of antimicrobial therapy (assuming no impact on outcome). The average daily cost of antimicrobial to treat carbapenem resistant Gram-negative bacteria was US$ 172 (US$ 110–235.3) versus US$ 28.3 (US$ 7.7–48.9) to treat carbapenem susceptible Gram-negative bacteria. Based on current data on the aetiology and resistance patterns of bacteria causing VARI [13], the proportion of VARI caused by carbapenem-resistant organisms was 16.4% (corresponding to 5,633 VARI episodes per year). However the costs of antimicrobial treatment alone of these episodes amounted to US$ 11,733,107, accounting for to 29% of the total national cost of VARI management.

**Fig 4:**
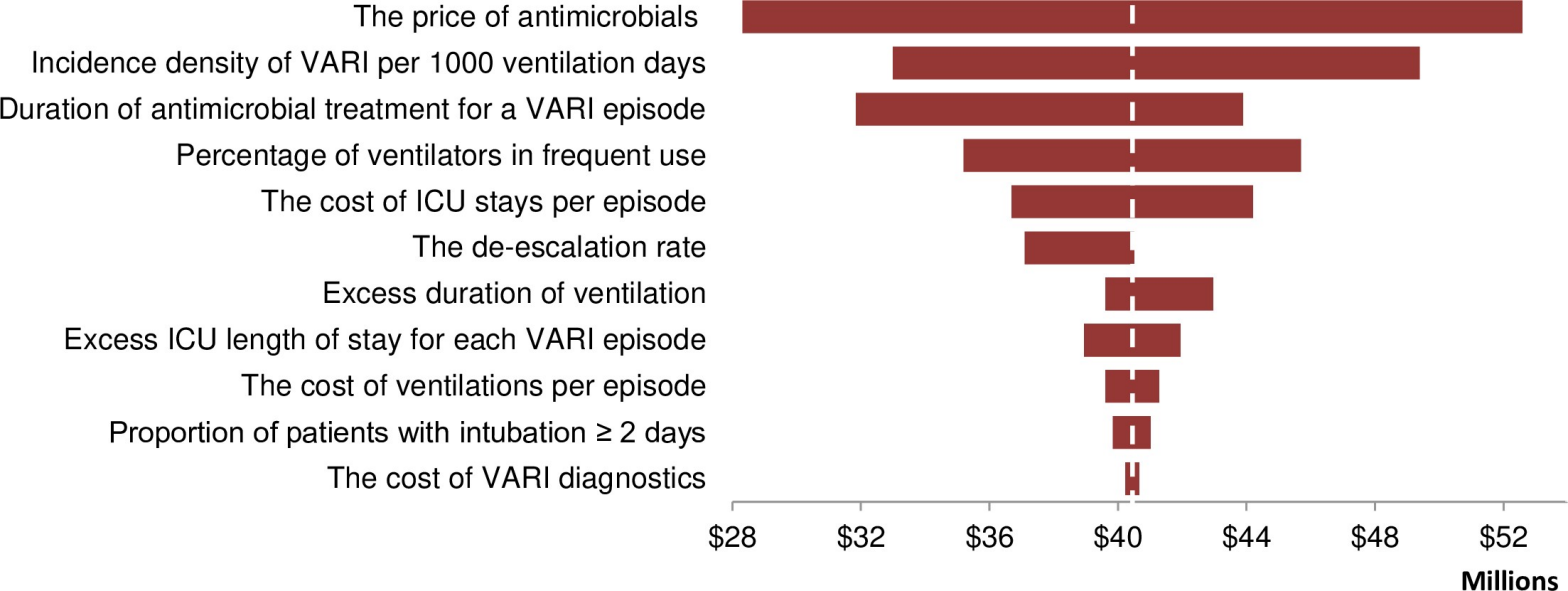
Sensitivity of modelled total costs associated with ventilator associated respiratory infection (VARI) diagnosis and treatment in Vietnam.

The tornado diagram (Fig 4) shows that the greatest impact on the national cost of VARI management was through the price of antimicrobials and the number of VARI episodes (the national cost varied from US$ 28,307,360–52,587,578). The variance of VARI incidence density is associated with a smaller cost variance (from US$ 32,991,714–49,394,375). Variance in the costs of VARI diagnostics, ventilation duration and the proportion of patients with intubation for more than 2 days had minor effects (Fig 3).

## Discussion

Our study estimates the burden and excess direct medical cost of VARI management across the whole of Vietnam, a lower-middle income country with a high rate of antibiotic resistance and hospital acquired infections. We estimated the excess cost of VARI to the Vietnamese health care system to be tens of millions of US Dollars, a high proportion of which was due to the cost of antibiotics.

The estimation of VARI cost per case in our study is comparable to previous studies in Vietnam but significantly lower than in high-income settings [6, 7]. The excess cost of HAI in Vietnam was reported as US$ 865 in a tertiary neonatal intensive care unit (ICU) in 2008 [29] and US$ 1,131 in tertiary ICU in 2010 [30]. In a study of 4 ICUs in 3 Vietnamese tertiary referral hospitals from 2013 to 2015, the median ICU related costs associated with admission were US$ 4,723 for patients developing VARI over their stay and US$ 2,534 for those that did not [15], corresponding to a cost difference of US$ 2,189. The lack of distinction between the attributed cost before and after VARI is a limitation of a non-matched cohort study [15, 31].

In a recent systematic review of the estimated economic impact of HAI on the US health care system it was shown that the most costly HAI was central line–associated bloodstream infection (US$ 45,814 in 2012 US$ per case), followed by VAP (US$ 40,144; 95% CI 36,286–44,220) [7]. The regional or national excess cost for VAP in Europe is estimated to be around US$ 13,000–15,000 per episode [32, 33]. However, the differences in incomes and healthcare systems make between countries comparisons relatively unedifying. However, given the current Vietnamese per capita health expenditure of US$ 117 (in 2015) [34], the estimated cost attributed to VARI management (US$ 1174.9) is a high economic burden to the healthcare system in Vietnam (in comparison to the ratio of US per capita health expenditure to attributable cost of US$ 9,536/US$ 40,144 [7]. These differences in cost may be attributed to differences in costing and the healthcare systems (in Vietnam some healthcare costs are borne ‘out of pocket’ and these were not included as they are very hard to quantify), similarly some costs for hospital infrastructure are subsidised by the government in Vietnam and many European countries. However there are also substantially lower costs for human resources in Vietnam and a tendency to use fewer and cheaper disposable items and blood tests.

Our data showed high variability in antimicrobial prices and a high proportion of costs related to antimicrobials (51.1%). In a matched control study of VAP in US, the attributed cost of VAP was US$ 39,828 per episode whilst the cost of antibiotics contributed to US$ 9,367 (23.5%) of the total cost [35]. The findings in our study likely relate to disproportionately high antibiotic costs in Vietnam [36], very high antibiotic resistance levels and long courses of treatment in Vietnam.

Despite growing healthcare insurance coverage in Vietnam, these high costs of treatment are often not fully covered by insurance. Thus the high cost of VARI may contribute to putting patients and their families into severe debt and act as a barrier to accessing treatment for life threatening conditions [37]. Our study highlights the savings that could be made from even small gains in the reduction of VARI incidence and any safe reduction in the length of therapy for VARI. These are even without potential gains through reducing exposure of the patient and the CCU environment to broad spectrum antibiotics.

Our estimate does not include non-medical direct costs including other out-of-pocket costs incurred by the patients and families for travelling, food and accommodation during hospitalization and consultations. It also does not include indirect costs associated with loss of income and productivity due to absence from work and premature mortality. Few data on these societal costs are available in Viet Nam. One study estimated that whilst the costs to the health sector averaged US$ 190 and US$ 300 for treating pneumonia and meningitis respectively, in children under 5 years old admitted to a tertiary hospital, the total direct non-medical costs (transportation cost, lodging, soap, diapers, etc) ranged from US$ 50 to US$ 156 and the indirect costs (defined as the productivity loss of caregivers based on the number of working hours lost and monthly income) ranged from US$ 60 to US$ 140 for pneumonia and for meningitis respectively [38].

One systematic review including three matched cohort studies reported a short attributable ICU length of stay related to VAP (a mean of 6 days, 95% CI, 5.32–6.87), [39] whilst one individual participant data meta-analysis from randomised trials assessing VAP prevention measures showed the overall difference of ICU length of stay was 12 days between VAP and non-VAP patients [28]. One prospective study in Vietnam showed the difference of ICU stays for VAP and non-VAP was 11 days [15]. We selected 12 days for our model because it reflects the longer duration of antimicrobial therapy used in Vietnam.

As there are no data for antimicrobial de-escalation rate in Vietnam, we assumed that the de-escalation was similar to studies in US and Europe [22, 40]. This is likely to represent an overestimate of the proportion that are de-escalated, in part due to limited opportunities to do so because of the high rates of antimicrobial resistance. Our model showed that variation in the proportion of patients de-escalated has a small impact on the total cost of VARI management.

Our study has several limitations. Firstly, resistance rates are increasing and the etiology and susceptibility data used may already be out of date, leading to an under-estimate of the attributable costs. Secondly, as a result of the increasing need for critical care to provide for an aging population with an increasing prevalence of underlying non-communicable diseases, the number of ventilated days nationally may rise leading to underestimation of the number of VARI episodes, in turn underestimating the total national cost of treatment. Furthermore, the studies quoted have focused on the first episode of VARI, potentially underestimating the incidence through exclusion of subsequent episodes. Thirdly, the critical care usage, the capacity of investigations of HAI (including microbiological testing) and the antibiotic prescription are difficult to extrapolate across Vietnam, as a nationwide CCU registry and quality improvement systems have not been established. Therefore data on transfer of patients, proportion of patients ventilated for more than 48hrs and antimicrobial prescribing patterns may vary substantially across hospitals.

## Conclusions

Our study found that the direct medical cost for VARI in Vietnam is substantial. Interventions targeting these infections, such as the implementation of infection control and antimicrobial stewardship in critical care units, could provide direct cost savings in addition to their beneficial effect on the generation of antibiotic resistance and should be systematically implemented and evaluated.

## Supporting information

S1 Table Assumed aetiology of ventilator associated respiratory infections. (DOCX)

## Abbreviations

95% CI: 95% confidence interval
CCU: critical care unit (all units that provide mechanical ventilation beds, includes emergency departments)
CHE: current health expenditure
GNI: gross national income
HAI: Hospital acquired infection
ICU: Intensive care unit
IQR: Interquartile range
VAP: Ventilator associated pneumonia
VARI: Ventilator-associated respiratory infection
VAT: Ventilator-associated tracheobronchitis
USD: United State dollar
VND: Vietnam Dong
