# Correction: Excess direct hospital cost of treating adult patients with ventilator associated respiratory infection (VARI) in Vietnam

**DOI:** 10.1371/journal.pone.0208361

**Published:** 2018-11-27

**Authors:** Vu Quoc Dat, Vu Thi Lan Huong, Hugo C. Turner, Louise Thwaites, H. Rogier van Doorn, Behzad Nadjm

There are errors in the Funding section. The correct funding information is as follows: This work was funded by the Wellcome Trust through their Asia Programme core grant.
