# Supplementary material for: Excess direct hospital cost of treating adult patients with ventilator associated respiratory infection (VARI) in Vietnam
Source: PLoS One. 2018 Oct 31;13(10):e0206760. doi: 10.1371/journal.pone.0206760 (PMC6209379; doi:10.1371/journal.pone.0206760)
Supplement: S1 Table — (DOCX) [file pone.0206760.s001.docx]

**S1 Table. Assumed aetiology of ventilator associated respiratory infections**

| **Name of microorganisms** | **Proportion** |
| --- | --- |
| **No identification of pathogens** | **61.5%** |
| **Identification of pathogens** | **38.5%** |
| ***Enterobacteriaceae*** | **6.7%** |
| *Escherichia coli* | 1.3% |
| Non-susceptible to the third-generation cephalosporins and carbapenems | 5.4% |
| Susceptible to the third-generation cephalosporins and non-susceptible to carbapenems | 54.1% |
| Susceptible to third-generation cephalosporins and carbapenems | 40.5% |
| *Klebsiella pneumoniae* | 4.5% |
| Non-susceptible to the third-generation cephalosporins and carbapenems | 14.9% |
| Susceptible to the third-generation cephalosporins and non-susceptible to carbapenems | 56.8% |
| Susceptible to third-generation cephalosporins and carbapenems | 28.4% |
| *Klebsiella spp.* | 2.9% |
| Non-susceptible to the third-generation cephalosporins and carbapenems | 5.7% |
| Susceptible to the third-generation cephalosporins and non-susceptible to carbapenems | 62.3% |
| Susceptible to third-generation cephalosporins and carbapenems | 30.1% |
| Other *Enterobacteriaceae* | 3.3% |
| Non-susceptible to the third-generation cephalosporins and carbapenems | 12.5% |
| Susceptible to the third-generation cephalosporins and non-susceptible to carbapenems | 59.4% |
| Susceptible to third-generation cephalosporins and carbapenems | 28.1% |
| **Non-fermenters** | **21.8%** |
| *Acinetobacter baumannii* | 9.9% |
| Non-susceptible to carbapenems | 89.2% |
| Susceptible to carbapenems | 10.8% |
| *Pseudomonas aeruginosa* | 6% |
| Non-susceptible to the third-generation cephalosporins and carbapenems | 55.7% |
| Susceptible to the third-generation cephalosporins and non-susceptible to carbapenems | 44.3% |
| Susceptible to third-generation cephalosporins and carbapenems |  |
| *Acinetobacter spp.* | 3% |
| Non-susceptible to carbapenems | 89.2% |
| Susceptible to carbapenems | 10.8% |
| Other Gram-negative bacilli | 2.9% |
| **Gram-positive bacteria** | **3.8%** |
| *Staphylococcus aureus* | 1.8% |
| Methicillin-resistant *Staphylococcus aureus* (MRSA) | 75.7% |
| Methicillin-sensitive *Staphylococcus aureus* (MSSA) | 24.3% |
| *Staphylococcus spp.* | 0.7% |
| Non-susceptible to oxacillin | 36.4% |
| Susceptible to oxacillin | 63.6% |
| *Enterococcus spp.* | 0.7% |
| Vancomycin-resistant Enterococcus (VRE) | 57% |
| Vancomycin-susceptible Enterococcus (VSE) | 43.0% |
| *Streptococcus spp*. | 0.6% |
| **Fungi (Candida spp.)** | **0.9%** |

Adapted from Phu VD, Wertheim HF, Larsson M, Nadjm B, Dinh QD, Nilsson LE, et al. Burden of Hospital Acquired Infections and Antimicrobial Use in Vietnamese Adult Intensive Care Units. PloS one. 2016;11(1):e0147544.
